# Older patients with persistent postural-perceptual dizziness exhibit fewer emotional disorders and lower vertigo scores

**DOI:** 10.1038/s41598-022-15987-w

**Published:** 2022-07-13

**Authors:** Li Zhang, Weiwei Jiang, Lu Tang, Hongxing Liu, Feng Li

**Affiliations:** Department of Neurology, Nanjing Brain Hospital Affiliated to Nanjing Medical University, No. 264 Guangzhou Road, Gulou District, Nanjing, Jiangsu China

**Keywords:** Neurological disorders, Psychiatric disorders

## Abstract

The clinical characteristics of persistent postural-perceptual dizziness (PPPD) vary according to patient age and inducing factors. We aimed to analyze the differences in the clinical characteristics of PPPD with different patient age groups and different etiologies. A total of 122 PPPD patients hospitalized in the vertigo ward of Nanjing Brain Hospital from December 2018 to July 2021 were enrolled. According to whether dizziness symptoms were secondary to organic diseases, PPPD patients were divided into the primary (p-) and secondary (s-) PPPD groups; subgroups were created according to age including youth group, middle-aged group, older adults group 1 and older adults 2. We collected detailed data from each patients, including scores on the Dizziness Handicap Inventory (DHI), mental state and other clinical data. The ratio of males to females was 1:2. The prevalence of emotional disorders in the middle-aged group was the highest (67.57%) and that in the older adults groups was lower (48.08% in older adults group 1 and 8.70% in older adults group 2, P = 0.000). The proportion of p-PPPD patients with emotional disorders was significantly higher than that of s-PPPD patients (53.48% vs. 30.56%, P = 0.028). The average total DHI score in the middle-aged group was significantly higher than that in older adults group 2 (52.86 vs. 35.04, P = 0.032), and the Beck anxiety score in the middle-aged group was higher than that in older adults group 2 (38.89 vs. 27.65, P = 0.000). The middle-aged group had the highest proportion of women, the highest proportion of patients with emotional disorders and the highest vertigo score. The proportion of patients with emotional disorders and the vertigo scores were lower in the older adults groups.

## Introduction

Dizziness, vertigo and balance disorders are common complaints in daily clinical medical work. These symptoms seriously affect patients' quality of life and limit the ability to work. The lifetime prevalence of severe dizziness is between 17 and 30%, and the lifetime prevalence of vertigo is between 3 and 10%^1^; nearly 20% of people over 65 years of age often complain of dizziness^2^. Some of these patients describe dizziness in their medical history as a vague feeling, such as chronic dizziness or unsteadiness, halo effects and feelings that the ground is rising and falling. The Bárány Society calls this subjective dizziness, which is common in the clinic and often with unclear vestibular dysfunction, persistent postural-perceptual dizziness (PPPD). The common precipitating factors of PPPD are vestibular diseases, including peripheral vestibular diseases and central vestibular diseases, as well as other organic diseases and mental illnesses. Dizziness, unsteadiness, and some types of non-spinning vertigo are the primary symptoms of PPPD. Some people describe the feeling of dizziness as fullness, heaviness, or lightness in the head, inaccurate spatial orientation or difficulty in focusing, which is a non-motion symptom. Unsteadiness is a feeling of instability when standing or veering from side to side when attempting to walk in a straight line. Some patients describe the non-spinning vertigo as feeling similar to rocking, bouncing, waving, or bobbing. This feeling can be that of movement inside the head, movement of the whole head or body, or movement of the environment^3^. Generally speaking, older adults usually have more basic diseases and worse physical strength than young people. Especially in older adults populations that experience dizziness, dizziness and balance disorders are the most common complaints. They lead to reduced physical activity, worse lower limb function and a higher risk of falls^4^. However, a patient diagnosis of PPPD can often be complicated with emotional disorders, which have many influencing factors. Additionally, the age characteristics of peripheral vestibular disorders differ. Patients with dizziness in different age groups and different inducing factors may differ in clinical characteristics, including the proportion of basic diseases, the proportion of vestibular diseases, the proportion of emotional disorders, dizziness scores and dizziness characteristics. Therefore, analyzing the clinical characteristics of vertigo patients of different age and different inducing factors can provide a deeper understanding of the disease, which can inform treatment and rehabilitation programs. We grouped PPPD patients by etiology and age to clarify the typical demographic and clinical characteristics of PPPD and the potential differences between primary and secondary variants of dizziness.

## Methods

### Patients

Based on the international classification of vestibular disorders and the PPPD diagnostic criteria and exclusion criteria, a total of 122 PPPD patients hospitalized in the vertigo ward of Nanjing Brain Hospital from February 2018 to July 2021 were enrolled.

The inclusion criteria involved meeting the following five conditions at the same time: 1. There is dizziness, instability and non-spinning vertigo for more than 3 months with (1) a persistent fluctuation in symptoms, and (2) these symptoms do not have to last all day. 2: There are no specific inducing factors, but the condition can be exacerbated by (1) upright posture, (2) active or passive movement (independent of direction and posture), and (3) moving visual stimulation or complex visual patterns. 3. The condition is induced by acute/chronic/episodic vestibular diseases, other systemic diseases and psychological distress: (1) when induced by acute/paroxysmal diseases, the symptoms can initially be relieved or intermittent, but they transform into a persistent course of disease; (2) when induced by a chronic disease, the symptoms appear slowly and then gradually aggravate. 4. The symptoms cause significant distress and dysfunction. 5. Other diseases cannot better explain the symptoms.

The exclusion criteria were as follows: (1) dizziness caused by systemic disease (including Parkinson's disease, Alzheimer's disease, sleep apnea syndrome, etc.); (2) dizziness caused by toxic substances and drugs; (3) dizziness caused by various withdrawal syndromes, including abstinence; (4) schizophrenia or mania or the consumption of related psychiatric medications; and (5) inability to complete the test due to illiteracy, intellectual disability or other reasons.

### Standard protocol approvals, registrations, and patient consents

This study was approved by the local ethics committee and was performed in accordance with the Helsinki Declaration of 1975. The data are anonymous, and the requirement for informed consent was therefore waived.

### Methods

All the patients were from the vertigo ward of Nanjing Brain Hospital and met the diagnostic criteria of PPPD. The patient's detailed medical case characteristics (characteristics of vertigo/dizziness attacks, course of disease, accompanying symptoms, duration, inducing factors, attack and frequency, etc.), past history and personal history were collected. In terms of medical history, we focused on the history of central vestibular diseases, such as cerebral infarction, intracerebral hemorrhage, intracranial tumors and other diseases, as well as the history of peripheral vestibular diseases, such as benign paroxysmal positional vertigo (BPPV), vestibular neuritis (VN), vestibular migraine (VM), and vestibular paroxysmia (VP). A neurophysical examination and vertigo bedside physical examination were conducted. Then the doctor communicated with the patients, explained the follow-up examination and questionnaire survey, and finally the patients signed the notice and other documents. Then, the patients were individually administered questionnaires in a separate room, such as Dizziness handicap inventory (DHI), Activities-specific balance confidence (ABC) scale, Beck anxiety inventory and Patient health questionnaire-9 items (PHQ-9), which were conducted by trained doctors or nurses. The patients were examined for vestibular function within 24 h after admission, including the Vestibular ocular reflex (VOR) and Alternate binaural bithermal caloric test. Keep the patient quiet and conscious when completing the questionnaire. Other relevant examinations, such as brain magnetic resonance imaging (MRI), neck vascular ultrasound, transcranial Doppler (TCD), cardiac ultrasound, routine blood tests and blood biochemistry tests, were conducted based on the patient’s condition.

The patients were divided into the youth group (≤ 40 years old), middle-aged group (41–59 years old), older adults group 1 (60–75 years old), and older adults group 2 (≥ 75 years old) based on age, and then additionally were divided into the secondary-PPPD (s-PPPD) and primary-PPPD (p-PPPD) groups based on whether or not the PPPD was secondary to an organic disease.

Emotional disorders were diagnosed based on the medical history and medical records provided by the patients. If the patient had a high DHI or Beck score with no previous history of emotional disorders, we administered the Hamilton Anxiety/Depression Rating Scale and Zung's Self Rating Anxiety/Depression Scale; if the patient scored high on these scales, we asked psychiatrists to confirm the diagnosis after consultation.

We record the feelings of dizziness described by the patients in as much detail as possible; these included feelings of spinning (of the self or the environment), fullness, heaviness, lightness in the head, difficulty focusing, unsteadiness, rocking, bouncing, waving, or bobbing. After analyzing the descriptions, we divided feelings of dizziness into hree categories: rotational, unsteadiness, and lightheadness. Each patient could experience one or more of these feelings. For each patient, the feelings of dizziness was determined by the trained doctor in the vertigo ward.

We divided the duration of dizziness into four groups: less than 1 min, 1–60 min, 1–24 h and more than 24 h. The duration of dizziness was determined according to the longest duration of the most frequent spells of dizziness and was carried out by the trained doctor in the vertigo ward.

We recorded the accompanying symptoms, such as headache, nausea, phono-/photophobia, loss of hearing, and innitus, and then determined the proportion of these symptoms experienced in each group. We also accessed the basic diseases of patients, such as hypertension, type 2 diabetes mellitus, coronary atherosclerotic heart disease, and stroke, based on the medical history and medical records provided by the patients. Then we determined the proportion of different kinds of basic diseases in each group and the total proportion of basic diseases in each group.

### Relevant evaluation scales

#### Dizziness handicap inventory (DHI)

The DHI is widely used in the diagnosis and treatment of dizziness and balance disorders. The scale includes 25 items (0 = no, 2 = sometimes, 4 = yes) and evaluates the situation of the patients with dizziness within three subcategories: emotional (E; 9 items, 36 maximal score), functional (F; 9 items, 36 maximal score) and physical handicap (P; 7 items, 28 maximal score). The range of DHI scores is from 0 to 100. The patients are instructed by doctors to rate the items by themselves, where 0 means that the vertigo disease had no impact on the patient. The higher the overall score was, the more severe the influence of vertigo on the patients. Through clinical verification, it was found that DHI has good internal consistency (α = 0.920)^5^. We recorded the total DHI score and the scores on terms E, F and P for each patient.

#### Activities-specific balance confidence (ABC) scale

The ABC scale is a 16-item self-assessment scale designed to assess confidence in maintaining balance. The ABC scale ranges from 0 to 100%, and the score ranges from 0 to 160. The ABC scale evaluates the completion of daily activities, including walking around the house, bending down to pick up objects and getting on the bus. The ABC scale has good internal consistency (α = 0.96) and good test–retest reliability and results in a stable total score when retested within 2 weeks^6^. We recorded the ABC score (0 to 160) of each patient.

#### Beck anxiety inventory

This is a self-assessment scale with a total possible score of 63 points for 21 items. The items were rated on a 4-point scale to assess the degree of annoyance experienced by the subjects by a variety of anxiety symptoms. It is suitable for adults with anxiety symptoms^7^. The Beck anxiety inventory has high internal consistency and high test–retest correlation and shows good simultaneous validity. Beck anxiety inventory scores are highly correlated with scores on the Symptom Checklist-90 (SCL-90) anxiety scale, Hamilton anxiety scale (HAMA) and State Trait Anxiety Inventory (STAI)^8^. We recorded the scores on each item and the total score of each patient.

#### Patient health questionnaire-9 items (PHQ-9)

The PHQ-9 is a questionnaire for diagnosing depression, assessing severity and monitoring treatment response. The PHQ-9 shows no significant ethnic differences across multiple populations (α = 0.79–0.89) and is easy to use^9^. We recorded the scores on each item and the total score of each patient.

### Vestibular examination

#### Vestibular ocular reflex (VOR)

As assessed by vHIT (ICS Impulse), the patient's vision was fixed on a target 1.5 m in front of him or her and evaluated by performing 20° rotations around three planes with small amplitude, high acceleration and passive head rotation. At least 10 suitable head rotation pulses were recorded during each test. A vHIT gain of less than 0.7 was considered pathological. The vHIT gain asymmetry index was calculated as follows: (difference between ipsilateral and contralateral gain/sum of ipsilateral and contralateral gain) × 100%. A vHIT gain asymmetry index of more than 10% is considered pathological. We recorded the VOR values of the six semicircular canals of each patient to estimate their peripheral vestibular system function. Any semicircular canal with a VOR less than 0.7 was considered abnormal. The proportion of abnormal VOR in each group was recorded.

#### Alternate binaural bithermal caloric test

The peak slow phase velocity (SPV) was measured by video oculography (ICS Chartr 200 VNG/ENG); the patient was in the supine position with the head at 30° to horizontal, the ears were irrigated with cold air (24 °C) and hot air (50 °C), and the maximum nystagmus velocity within 90 s was recorded. Based on the Jongkees index formula, if the canal paresis (CP) value was greater than 25%, it was defined as abnormal. The proportion of abnormal results on the alternate binaural bithermal caloric test in each group was recorded.

### Statistics

Data were processed by SPSS 26.0 (IBM, Chicago, IL, USA). The measurement data that were normally distributed are expressed as the mean ± standard deviation (SD), and the comparison between groups was performed by one-way ANOVA, which requires homogeneity in statistical variance. If the variance was not uniform, differences were evaluated by the Kruskal–Wallis test. Categorical variables are expressed as frequencies and percentages. When comparing more than two groups or factors, we used the chi-square test. Correlations were measured with Pearson's r. If the frequency was less than 5, Fisher's exact test was used, and P < 0.05 was considered to be statistically significant.

### Ethics approval

This study was approved by the Ethics Committee of Nanjing Brain Hospital and exempted from the application for informed consent.

## Results

### Basic characteristics

**Figure 1:**
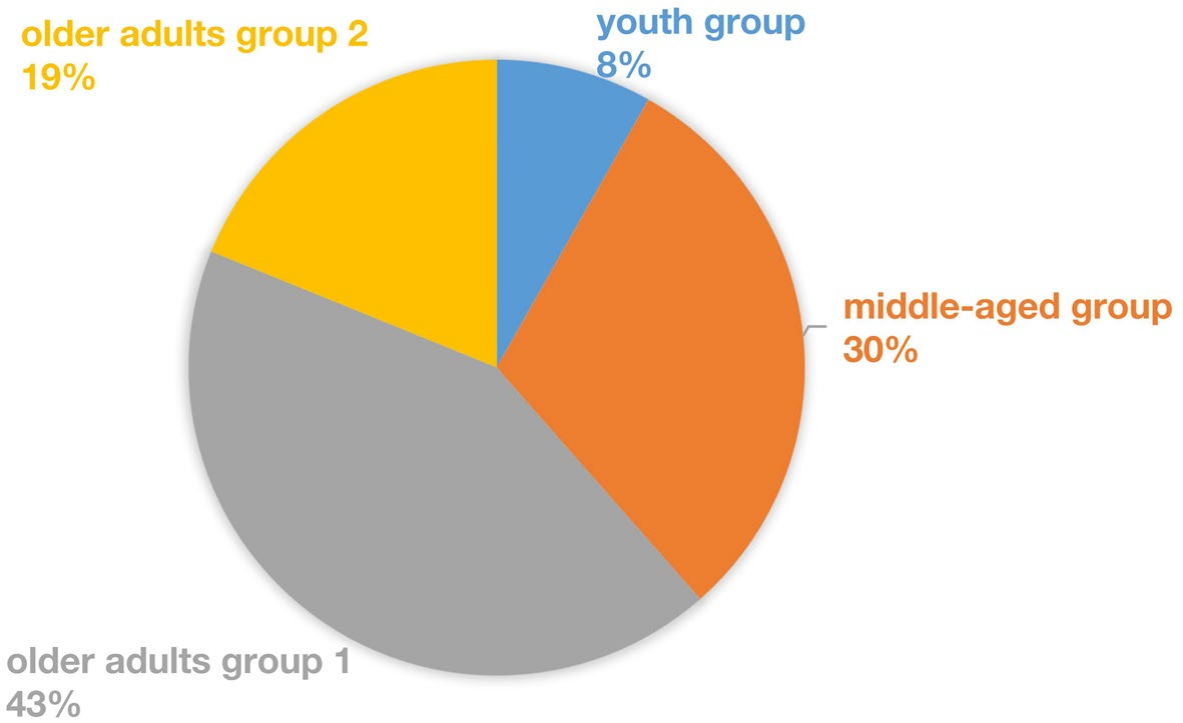
Age composition ratio of PPPD patients.

**Figure 2:**
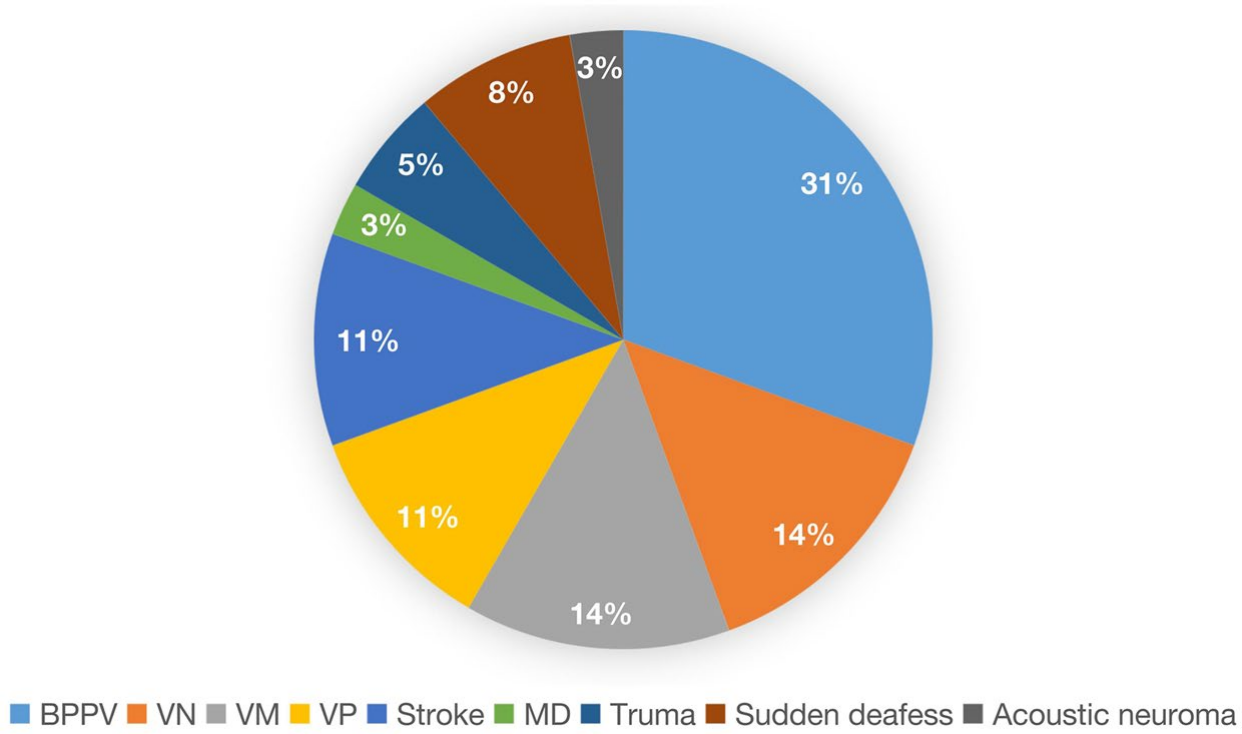
Proportion of secondary diseases in patients with s-PPPD.

**Figure 3:**
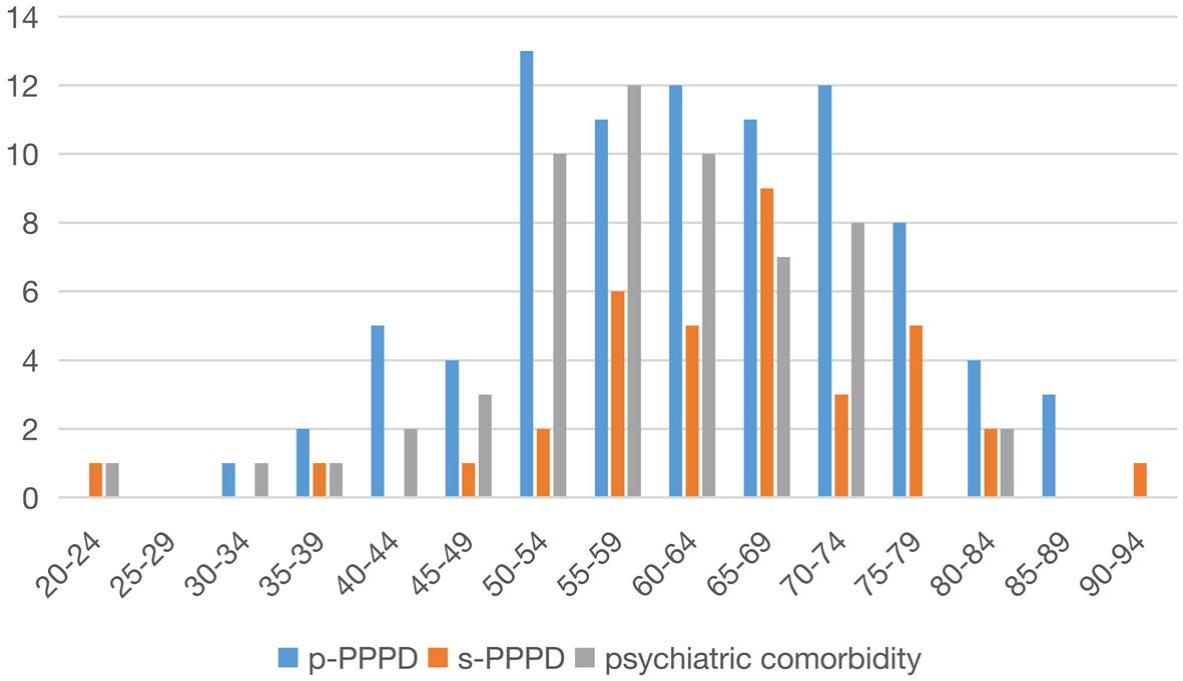
Number of patients with p-PPPD, s-PPPD and psychiatric comorbidity in each age group.

A total of 122 patients with PPPD, 86 patients with p-PPPD (70.49%) and 36 patients with s-PPPD (29.51%), were included, and the sample comprised 41 males (33.6%) and 81 females (66.4%), with a male to female ratio of 1:2, was an average age of 62.76 years and had an average course of 3.56 years. Among them, 10 patients were younger than 44 years old (youth group), 37 patients were 45 to 59 years old (middle-aged group), 52 patients were 50 to 64 years old (older adults group 1), and 23 patients were older than 65 years old (older adults group 2) (Fig. 1). S-PPPD was mostly secondary to organic diseases such as BPPV, VN, VM, VP, Meniere's disease (MD), stroke, sudden deafness, truma and acoustic neuroma (Fig. 2). The age histogram showed that the age range with the highest levels of anxiety and the highest proportion of p-PPPD patients was 50–74 years, the highest proportion of s-PPPD patients was in the 55–79 years age range, and the peak age of the s-PPPD patients was 65–69 years (Fig. 3).

### Emotional disorders

This study showed that 57 patients (46.72%) were diagnosed with emotional disorders at discharge, including 56 patients with anxiety (45.90%) and 18 patients with depression (14.75%). The prevalence of emotional disorders was highest in the middle-aged group, at 67.57%. The proportion of emotional disorders was lower in the older adults group. With increasing age, the proportion of emotional disorders in older adults group 2 was lower than that in older adults group 1 and the other groups (P < 0.001). The proportion of middle-aged patients with p-PPPD complicated with emotional disorders was as high as 75%, which was significantly higher than that in other subgroups (P < 0.001). The proportion of patients with emotional disorders in the p-PPPD group was markedly higher than that in the s-PPPD group (53.48% vs. 30.56%, p = 0.028); the proportion of patients with anxiety disorders was also significantly different (p = 0.030), although there was no difference in the proportion of patients with depression (Table 1).

**Table 1:** Baseline characteristics of patients.

	Youth group (10)		Middle-aged group (37)		Older adults group 1 (51)		Older adults group 2 (24)		All (122)	p	p-PPPD (86)	s-PPPD (36)	p	Youth (10)	Middle-aged (37)	Older adults 1 (51)	Older adults 2 (24)	p
	p-PPPD (8)	s-PPPD (2)	p-PPPD (28)	s-PPPD (9)	p-PPPD (35)	s-PPPD (16)	p-PPPD (15)	s-PPPD (9)										
Age in years (mean, SD)	39.75 ± 3.81	31.00 ± 11.31	53.21 ± 3.50	55.44 ± 3.64	66.46 ± 3.11	66.23 ± 3.19	79.73 ± 3.93	79.88 ± 5.59	62.76 ± 12.36	0.000	61.98 ± 12.25	64.61 ± 12.58	0.285	38.00 ± 6.26	53.76 ± 3.62	66.38 ± 3.80	79.78 ± 4.45	0.000
Gender (% female)	6 (75.00)	2 (100.00)	16 (57.14)	7 (77.78)	22 (62.86)	12 (70.59)	9 (60.00)	7 (87.50)	81 (66.39)	0.752	53 (61.62)	28 (77.78)	0.097	8 (80.00)	23 (62.16)	34 (34.62)	16 (48.48)	0.794
Disease duration in years (mean, SD)	2.84 ± 1.99	0.50 ± 0.00	1.90 ± 2.18	3.13 ± 4.46	3.72 ± 5.28	5.38 ± 7.90	5.68 ± 5.06	2.75 ± 1.28	3.56 ± 4.86	0.200	3.39 ± 4.35	3.96 ± 5.97	0.557	2.38 ± 2.01	2.20 ± 2.88	4.23 ± 6.23	4.66 ± 4.34	0.123
Emotional disorders	4 (50.00)	1 (50.00)	21 (75.00)	4 (44.44)	20 (57.14)	5 (29.41)	1 (6.67%)	1 (12.50)	57(46.72)	0.000	46 (53.48)	11 (30.56)	0.028	5 (50.00)	25 (67.57)	25 (48.08)	2 (8.70)	0.000
Depressive disorder (%)	2 (25.00)	1 (50.00)	7 (25.00)	2 (22.22)	5 (14.29)	1 (5.88)	0 (0.00)	0 (0.00)	18 (14.75)	0.114	14 (16.28)	4 (11.11)	0.582	3 (10.00)	9 (24.32)	6 (11.54)	0 (0.00)	0.016
Anxiety disorder (%)	4 (50.00)	1 (50.00)	21 (75.00)	4 (44.44)	19 (54.29)	5 (29.41)	1 (6.67%)	1 (12.50)	56 (45.90)	0.000	45 (52.33)	11 (30.56)	0.030	5 (50.00)	25 (67.57)	24 (46.15)	2 (8.70)	0.000
**Type of vertigo**																		
Rotational (%)	2 (25.00)	2 (100.00)	5 (17.86)	5 (55.55)	9 (25.71)	7 (41.18)	4 (26.67)	3 (37.50)	37 (30.33)	0.148	20 (23.25)	17 (47.20)	0.011	4 (40.00)	10 (27.23)	16 (30.77)	7 (30.43)	0.882
Unsteadiness (%)	4 (50.00)	0 (0.00)	8 (28.57)	2 (22.22)	12 (34.29)	7 (41.18)	5 (33.33)	5 (62.50)	43 (35.24)	0.607	29 (33.72)	14 (38.89)	0.586	4 (40.00)	10 (27.23)	19 (36.54)	10 (43.48)	0.573
Lightheadedness (%)	6 (75.00)	2 (100.00)	25 (89.29)	4 (44.44)	27 (77.14)	9 (52.94)	13 (86.67)	6 (75.00)	92 (75.41)	0.054	71 (82.56)	21 (58.33)	0.006	8 (80.00)	29 (78.38)	36 (69.23)	19 (82.61)	0.613
**Length of vertigo episodes**																		
< 1 min (%)	0 (0.00)	1 (50.00)	4 (16.67)	3 (33.33)	2 (5.71)	2 (11.76)	0 (0.00)	1 (12.50)	13 (10.66)	0.087	6 (6.98)	7 (19.44)	0.055	1 (10.00)	7 (18.92)	4 (7.69)	1 (4.34)	0.290
1–60 min (%)	0 (0.00)	0 (0.00)	3 (10.71)	1 (11.11)	7 (20.00)	4 (23.53)	3 (20.00)	2 (25.00)	20 (16.39)	0.786	13 (15.12)	7 (19.44)	0.596	0 (0.00)	4 (10.81)	11 (21.15)	5 (21.74)	0.282
1–24 h (%)	5 (62.50)	1 (50.00)	12 (42.86)	3 (33.33)	14 (40.00)	6 (35.29)	7 (46.67)	3 (37.50)	51 (41.80)	0.946	38 (44.19)	13 (36.11)	0.430	6 (60.00)	15 (40.54)	20 (38.46)	10 (43.48)	0.665
> 24 h (%)	3 (37.50)	0 (0.00)	9 (32.14)	2 (22.22)	12 (34.29)	5 (29.41)	5 (33.33)	2 (25.00)	38 (31.15)	0.996	29 (33.72)	9 (25.00)	0.397	3 (10.00)	11 (29.73)	17 (32.69)	7 (30.43)	0.990
**Accompanying symptoms**																		
Headache (%)	0 (0.00)	1 (50.00)	2 (7.14)	1 (11.11)	3 (8.57)	2 (11.76)	0 (0.00)	0 (0.00)	9 (7.38)	0.214	4 (4.65)	5 (13.89)	0.122	1 (10.00)	3 (8.11)	5 (9.62)	0 (0.00)	0.491
Nausea (%)	4 (50.00)	1 (50.00)	11 (39.29)	5 (55.55)	9 (25.71)	7 (41.18)	4 (26.67)	2 (25.00)	43 (35.24)	0.587	28 (32.56)	15 (41.67)	0.407	5 (50.00)	16 (43.24)	16 (30.77)	6 (26.09)	0.356
Phono-/photophobia (%)	2 (25.00)	1 (50.00)	8 (28.57)	1 (11.11)	8 (22.22)	7 (41.18)	2 (13.33)	0 (0.00)	29 (23.77)	0.305	20 (23.25)	9 (25.00)	1.000	3 (10.00)	9 (24.32)	15 (28.85)	2 (8.70)	0.249
Loss of hearing (%)	0 (0.00)	0 (0.00)	2 (7.14)	0 (0.00)	0 (0.00)	2 (11.76)	2 (13.33)	0 (0.00)	6 (4.92)	0.303	4 (4.65)	2 (5.56)	1.000	0 (0.00)	2 (5.41)	2 (3.85)	2 (8.70)	0.801
Tinnitus (%)	2 (25.00)	0 (0.00)	9 (32.14)	1 (11.11)	5 (14.29)	5 (29.41)	2 (13.33)	1 (12.50)	25 (20.49)	0.628	18 (20.93)	7 (19.44)	1.000	2 (25.00)	10 (27.23)	10 (19.23)	3 (13.04)	0.631
**Basic disease**																		
Hypertension	2 (25.00)	0 (0.00)	10 (35.71)	0 (0.00)	24 (47.05)	9 (52.94)	12 (80.00)	5 (62.50)	62 (50.82)	0.000	48 (55.81)	14 (38.89)	0.113	2 (25.00)	10 (27.23)	33 (64.71)	17 (70.83)	0.000
Type 2 diabetes mellitus	0 (0.00)	0 (0.00)	3 (10.71)	1 (11.11)	4 (7.84)	2 (11.76)	4 (26.67)	4 (50.00)	18 (14.75)	0.157	11 (12.79)	7 (19.44)	0.404	0 (0.00)	4 (10.81)	6 (11.54)	8 (33.33)	0.038
Coronary atherosclerotic heart disease	0 (0.00)	0 (0.00)	1 (3.57)	0 (0.00)	4 (7.84)	0 (0.00)	4 (26.67)	2 (25.00)	11 (9.01)	0.092	9 (10.47)	2 (5.56)	0.504	0 (0.00)	1 (2.70)	4 (7.69)	6 (26.09)	0.025
Stroke	0 (0.00)	0 (0.00)	7 (25.00)	1 (11.11)	23 (45.1)0	7 (41.18)	7 (46.67)	7 (87.50)	52 (42.62)	0.000	37 (43.02)	15 (41.67)	1.000	0 (0.00)	8 (21.62)	30 (58.82)	14 (58.33)	0.000

### Dizziness characteristics

The feeling of lightheadedness was the main characteristic of PPPD patients. A total of 75.41% of patients had this characteristic, followed by unsteadiness and rotation, which were experienced by 35.24% and 30.33% of the patients, respectively. There were no significant differences in the proportion of different forms of dizziness among the 4 age groups, but the proportion of rotation in the s-PPPD group was significantly higher than that in the p-PPPD group (47.20% vs. 23.25%, p = 0.011) (Table 1).

### Dizziness scores

**Figure 4:**
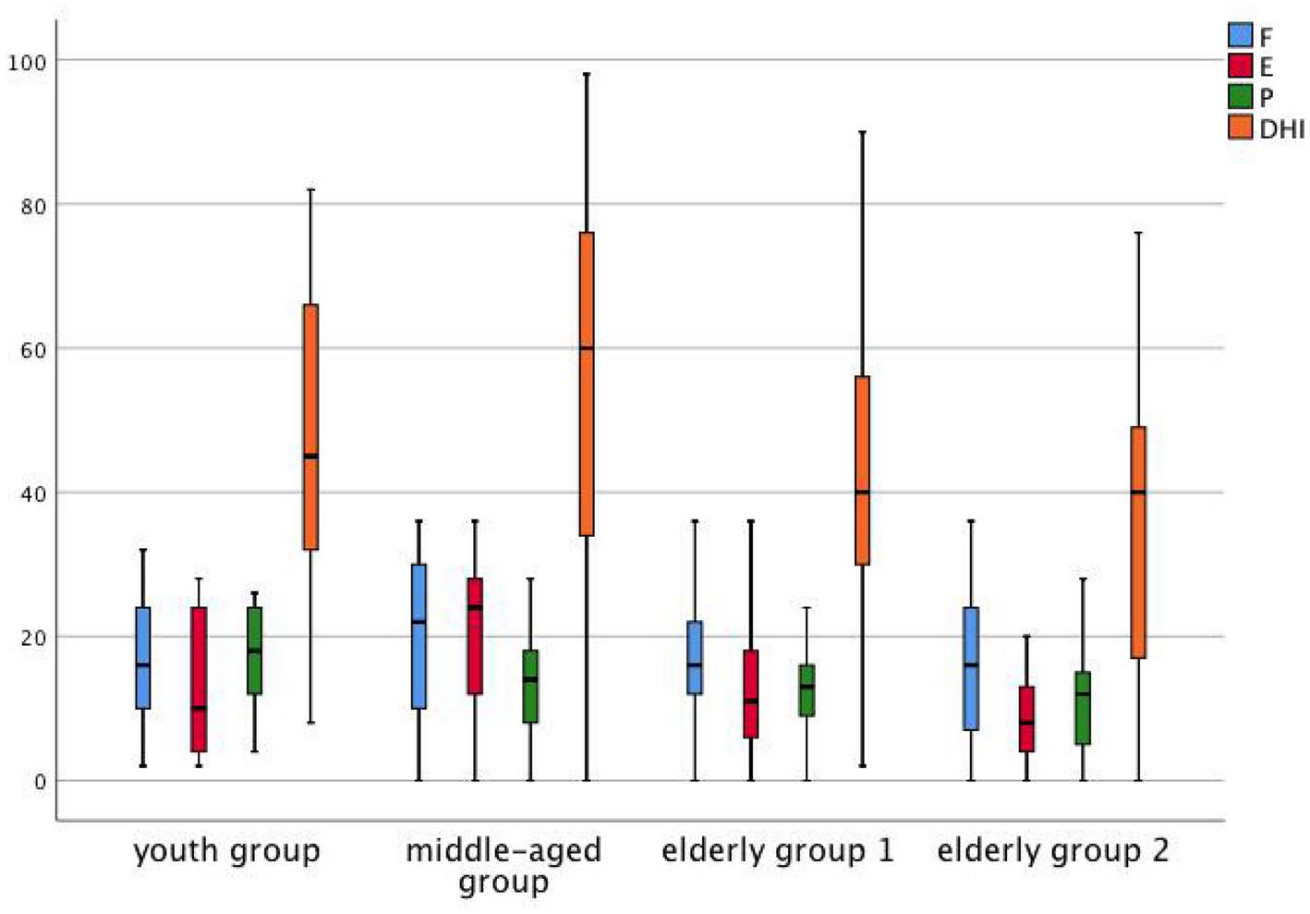
Box plots of DHI score in each group.

**Figure 5:**
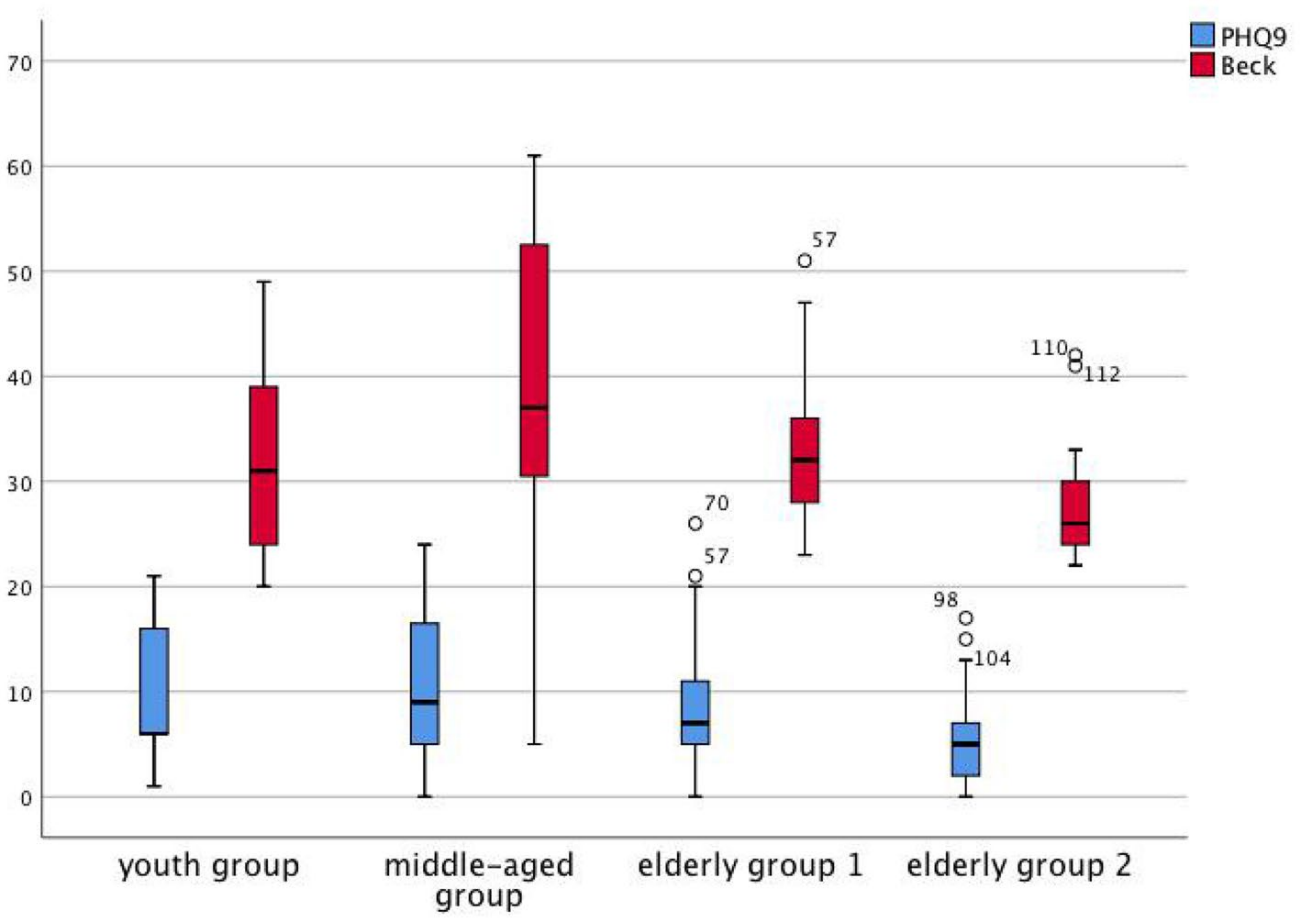
Box plots of PHQ-9 and Beck score in each group.

Among the 4 age groups, the average total DHI score in the middle-aged group was 52.86, which was the highest, and the average score in older adults group 2 was 35.04, which was the lowest (p = 0.032); the average E score in the middle-aged group was 19.08, and the average E score in older adults group 2 was 8.35, which is the lowest (P = 0.002). The P score in the s-PPPD group was higher than that of the p-PPPD group (P = 0.024), but there was no significant difference in the total DHI score (Fig. 4) The Beck anxiety scale score in the middle-aged group was significantly higher than those in the other groups. The scores of the s-PPPD patients in the middle-aged group, p-PPPD patients in the middle-aged group and s-PPPD patients in the youth group were the highest, and the scores of older adults group 2 were the lowest (p < 0.001) (Fig. 5).

### Vestibular examination

A total of 30 patients assessed with the vHIT examination had abnormalities (24.59%), and there were no significant differences in this abnormal rate among the 4 age groups, but the abnormal rate in the s-PPPD group was significantly higher than that in the p-PPPD group (44.44% vs. 16.28%, respectively, p = 0.001). A total of 100 alternate binaural bithermal caloric tests were completed. Twenty-two patients failed to complete the examination due to intolerance, and 72 patients (72%) showed abnormalities (Table 2).

**Table 2:** Vertigo related score and examination.

	Youth group (10)		Middle-aged group (37)		Older adults group 1 (51)		Older adults group 2 (24)		All 122	p	p-PPPD (86)	s-PPPD (36)	p	Youth (10)	Middle-aged (37)	Older adults 1 (51)	Older aultes 2 (24)	p
	p-PPPD (8)	s-PPPD (2)	p-PPPD (28)	s-PPPD (9)	p-PPPD (35)	s-PPPD (16)	p-PPPD (15)	s-PPPD (9)										
Total DHI score (mean, SD)	38.50 ± 19.53	81.00 ± 1.41	51.86 ± 26.42	56.00 ± 37.95	38.69 ± 20.22	48.47 ± 18.62	33.73 ± 17.14	37.50 ± 26.59	44.34 ± 24.19	0.023	42.09 ± 22.69	49.72 ± 27.02	0.112	47.00 ± 24.86	52.86 ± 29.10	41.88 ± 20.08	35.04 ± 20.38	0.032
Emotional subscore	10.00 ± 9.50	26.00 ± 2.83	18.86 ± 10.40	19.78 ± 14.68	12.82 ± 9.59	12.75 ± 9.90	7.60 ± 5.96	9.75 ± 6.96	13.67 ± 10.15	0.001	13.28 ± 9.78	14.61 ± 11.07	0.511	13.20 ± 10.80	19.08 ± 11.37	12.27 ± 8.92	8.35 ± 6.26	0.000
Functional subscore	13.50 ± 6.57	31.00 ± 1.41	19.71 ± 11.13	22.22 ± 14.44	15.60 ± 9.87	19.41 ± 6.59	16.27 ± 9.82	15.50 ± 13.64	17.75 ± 10.38	0.192	16.86 ± 10.11	19.89 ± 10.85	0.142	17.00 ± 9.39	20.32 ± 11.85	16.85 ± 9.04	16.00 ± 10.99	0.34
Physical subscore	15.00 ± 7.93	24.00 ± 0.00	13.28 ± 7.25	14.00 ± 9.64	11.09 ± 6.48	16.23 ± 5.29	9.73 ± 5.99	12.00 ± 9.80	12.89 ± 7.28	0.041	11.93 ± 6.87	15.17 ± 7.80	0.024	16.80 ± 7.96	13.46 ± 7.76	12.77 ± 6.53	10.52 ± 7.39	0.134
ABC	126.75 ± 28.44	110.50 ± 4.95	105.07 ± 39.78	90.67 ± 63.23	111.15 ± 36.70	102.50 ± 41.29	102.86 ± 36.94	104.38 ± 38.60	106.58 ± 39.66	0.751	109.20 ± 37.10	100.34 ± 45.13	0.269	123.50 ± 26.06	101.56 ± 45.98	108.33 ± 38.05	103.41 ± 36.63	0.454
Beck Anxiety	30.88 ± 9.68	39.50 ± 13.43	39.107 ± 12.88	38.22 ± 12.80	32.29 ± 6.83	31.70 ± 6.85	27.87 ± 4.93	27.25 ± 7.03	33.36 ± 9.85	0.002	33.60 ± 10.01	32.78 ± 9.58	0.674	32.60 ± 10.30	38.89 ± 12.69	32.10 ± 6.78	27.65 ± 5.59	0.000
PHQ-9	8.25 ± 5.73	13.50 ± 10.61	10.68 ± 6.34	12.00 ± 7.97	9.20 ± 5.44	7.47 ± 6.10	5.40 ± 4.36	5.63 ± 5.80	8.81 ± 6.14	0.046	8.93 ± 5.80	8.53 ± 6.96	0.743	9.30 ± 6.55	11.00 ± 6.67	8.63 ± 5.66	5.48 ± 4.78	0.008
FRQ	4.13 ± 2.30	5.50 ± 3.54	5.44 ± 2.38	4.89 ± 2.32	5.00 ± 2.08	5.00 ± 2.33	5.33 ± 2.10	5.88 ± 2.80	5.14 ± 2.26	0.861	5.11 ± 2.20	5.21 ± 2.41	0.830	4.40 ± 2.41	5.29 ± 2.34	5.00 ± 2.14	5.55 ± 2.35	0.563
Abnormal vHIT (%)	0 (0.00)	1/2 (50.00)	6 (25.55)	3 (33.33)	5 (14.29)	8 (47.06)	3 (20.00)	4 (50.00)	30 (24.59)	0.046	14 (16.28)	16 (44.44)	0.001	1 (10)	9 (24.32)	13 (25.00)	7 (30.43)	0.731
Alternate binaural bithermal caloric test(abnormal/finished, %)	5/6 (83.33)	1/2 (50.00)	12/20 (60.00)	6/9 (66.67)	23/29 (79.31)	14/17 (82.35)	5/10 (50.00)	6/7 (85.71)	72/100 (72.00)	0.400	45/65 (69.23)	27/35 (77.14)	0.487	6/8 (75.00)	18/29 (62.07)	37/46 (80.43)	11/17 (64.71)	0.311

### s-PPPD as a secondary factor

A total of 31% patients had PPPD secondary to BPPV, followed by VN, vestibular migraine (VM), vestibular paroxysmia (VP) and stroke. The first three conditions accounted for nearly 60% of the patients (Fig. 5).

### Basic diseases

The top four basic diseases were hypertension (62 patients), stroke (52 patients), type 2 diabetes mellitus (11 patients) and coronary heart disease (9 patients). Across the 4 age groups, the incidence of basic diseases increased with age (Table 1).

## Discussion

The data from our center showed that the proportion of patients with p-PPPD and s-PPPD was 1:2.4, and the proportion of men and women was 1:2. The number of patients with p-PPPD was significantly greater than that with s-PPPD, and the number of women was significantly greater than that of men. There were more female patients in the s-PPPD group. Many studies have confirmed that p-PPPD and female patients are dominant in PPPD. Retrospective studies have shown that p-PPPD patients account for more than s-PPPD patients (55% vs. 45%) and that there are more female patients with PPPD (female:male = 56%:44%), especially in the s-PPPD group (64%)^10^. Women were slightly predominant in this PPPD cohort, especially in the s-PPPD group, which may be partly due to the higher prevalence of some organic vertigo diseases among women, such as VM^11^, MD^12^ and BPPV^13^. In addition to functional dizziness, somatoform pain disorder^14^ and nonepileptic seizures^15^ are experienced more often by female patients. Somatoform dysfunction has been shown to be correlated with sex.

Our study showed that the average age of onset of PPPD was 62.76 years old, which was significantly older than the age reported in previous studies. The percentage of patients over 60 years old was 61.48%, and the percentage of patients over 75 years old was 18.85%, while the proportion of young patients was low, with only 8.20% of patients under 44 years old. The reason may be that all the patients included in the study were hospitalized, so it is possible patients with more serious conditions were selected for. Young and middle-aged patients generally have a lower willingness to be hospitalized due to busy work schedules and taking care of their families; in contrast, older adults patients have more time and more basic diseases than young patients, so they have a higher willingness to be hospitalized. This study showed that the age group with the highest frequency of patients with p-PPPD was between 50 and 74 years old, and there were no significant differences in the number of patients across all ages. The age group with a high frequency of patients with s-PPPD was between 55 and 79 years old, but it reached the peak at 65–69 years old. Although there was no significant difference in the average age of patients between the two groups, we found that the patients in the p-PPPD group were younger.

Chinese studies have shown that the age of onset of PPPD was 40–60 years^16^ and is greater for s-PPPD^10^; in particular, the average onset age for s-PPPD was 52 years^17^ versus 42 years for p-PPPD^18^, considering that the incidence of vestibular diseases such as BPPV in older adults patients was higher than that in other age groups^13^. Although p-PPPD and s-PPPD can occur at any age, retrospective studies have shown that the peak of the age distribution curve is 50–55 years and that p-PPPD patients have another early peak at approximately 30 years of age^10^. Among the patients with internal medical diseases, female patients with somatoform disorders were more common, and the most common onset age was between 20 and 40 years; 25% of somatoform disorders are complicated with anxiety, and 11% are complicated with depression^19^, which is related to the younger onset age of p-PPPD patients.

This study showed that there was no significant difference in the single attack durations of dizziness and accompanying symptoms (headache, nausea/vomiting, photophobia/fear of sound, hearing loss and tinnitus). The proportion of patients experiencing a sense of rotation in the s-PPPD group was significantly higher than that in the p-PPPD group. In addition, there were no significant differences in the constituent ratios of dizziness types among the patients in each group. Considering that s-PPPD is mostly secondary to organic diseases such as BPPV, VN, VM and VP, visual rotation and other symptoms often occur with acute attacks of vestibular diseases, and similar symptoms remain with the gradual development of chronicity of the disease. However, more than 20% of patients with p-PPPD also had a sense of rotation. Considering that the sense of rotation is a manifestation of many somatization symptoms caused by psychological diseases, the sense of rotation is a clinical symptom related to the nervous system. Therefore, in clinical diagnosis, if patients have more complaints and cannot fully meet the diagnostic criteria based on a single symptom, psychological diseases or combined psychological diseases should be considered. Studies have shown that the proportion of rotation in patients with s-PPPD is 67%, while that in patients with p-PPPD is 34%^10^. In our study, the proportion of sense of rotation was low. It may have been that the patients answered questions more carefully and had clinicians' auxiliary explanations and guidance during completion of the questionnaire survey. Meanwhile, the average age of patients in this study was older, which was caused by the difference in age composition. The proportion of patients with dizziness attacks lasting less than 1 min in the s-PPPD group was greater than that in the p-PPPD group (19.44% vs. 6.98%), which is consistent with peripheral vestibular disease in s-PPPD patients as the main cause, but the p value was 0.055, which did not reach statistical significance.

This study showed that the average DHI score in the middle-aged group was the highest, while that in older adults group 2 was the lowest, and the same E-score results were obtained. The Beck anxiety scale score in older adults group 2 was also significantly lower than that in the middle-aged group. Combined with the fact that the prevalence of emotional diseases in the middle-aged group was significantly higher than that in the older adults groups, we see that with increasing age, the proportion of PPPD patients with psychiatric disorders gradually decreased, and the age distribution peak of psychiatric disorders was between 50 and 74 years, which coincided with the age peak for p-PPPD. Another study showed that among older adults patients with dizziness and vertigo, the anxiety scores based on the vertigo severity score (VSS), autonomic-anxiety on the VSS (VSS-A), vertigo-balance on the VSS (VSS-V) and the hospital anxiety and depression scale (HADS) score were significantly lower than those among young patients and that the measures of independence, autonomy and balance capacity were improved after 6 months of treatment, although the anxiety scores did not significantly change^20^. It is suggested that the influence of emotional factors on dizziness and vertigo in older adults patients is significantly less than that in young and middle-aged patients.

The average P and F scores of older adults patients were lower than those of young and middle-aged patients. Although the differences were not statistically significant, they at least show that the degree of subjective dysfunction was not more serious in older adults patients than in young patients. Considering that older adults patients have a higher probability of basic diseases and a higher proportion of previous stroke, they theoretically should have higher scores regarding physical aspects and dysfunction, but the actual situation is surprising. A recent study has shown that the symptoms of PPPD are common in the population. The visual vertigo simulation scale (VVAS) and situational characteristics questionnaire (SCQ) scores of more than 2000 volunteers were collected, and subclinical symptoms of PPPD were shown to be very common in the general population. The results showed that the severity of dizziness as assessed by the VVAS scores and SCQ scores were negatively correlated with age; therefore, PPPD preexists in the population and is not just the result of vestibular injury. Atypical visual vestibular processing systems tend to cause visual vertigo in some people, which can be exacerbated if vestibular injury (or more extensive injury) occurs^21^. Because the main research subjects in that study were online questionnaire respondents and college students, it is possible that younger patients were differentially selected for and that there was a certain age limit, but the conclusion was that older patients still have lower scores.

For patients with dizziness, research has shown that the causes among older adults patients were mostly degenerative diseases, such as multisensory deficits and central vertigo (stroke); among middle-aged patients, dizziness was shown to be mostly caused by somatoform disorders, phobic dizziness, secondary somatoform disorders and so on^20^. Other studies have found that the inducing factors of s-PPPD, from greatest to lowest influence, are BPPV, VM, unilateral vestibulopathy, trauma, MD, stroke, syncope and so on^10^. Based on our data, the proportion of s-PPPD in the older adults group was higher than that in the youth group and the middle-aged group. Among all s-PPPD patients, 31% were secondary to BPPV, followed by VN, VM, VP and stroke. BPPV is still the main inducing disease of s-PPPD. The causes of s-PPPD in the older adults group were mainly BPPV, VM and stroke. The main causes of s-PPPD in the middle-aged group were BPPV and VN. Our study shows that although the proportion of BPPV in older adults patients is high, the proportion of mental disease in older adults patients is lower than that in youth and middle-aged groups. Epidemiological studies have shown that the peak age of anxiety is in the early stage of adulthood, and the incidence and prevalence decrease with age^22^. Another study showed that although older adults patients with generalized anxiety disorder have less severe symptoms than younger patients, they have more sleep disorders, a higher incidence of depression and a higher degree of depression^23^. The survey results showed that we underestimate the prevalence of anxiety in the older adults population. The reason may be that older adults individuals are unwilling to reveal their anxiety symptoms, which leads to a decline in the evaluation of anxiety in these individuals and may result in recall bias^24^. Some dizzy patients who can walk independently without obvious imbalance symptoms are often ignored. BPPV in older adults patients sometimes shows only the feeling of instability rather than the sense of rotation in youth and middle-aged patients. Due to their mild symptoms, the treatment rate is low. When they see a doctor for long-term dizziness, it is considered secondary PPPD caused by BPPV, and the symptoms of patients can be significantly improved through rehabilitative treatment^25^. The main inducing factor of BPPV for s-PPPD is mainly related to the high incidence rate of BPPV in the population. Research has shown that the probability of secondary mental illness in BPPV is low^26^. Prospective studies have found that the probability of VN combined with mental illness is higher than the probability of BPPV combined with mental illness, and VM patients were more likely to have chronic dizziness and mental illness^27^. A prospective follow-up study of BPPV, VM, VN and MD patients revealed that only VM patients had a higher incidence rate of mental illness. A history of positive mental disorders was a strong predictor of the development of reactive mental disorders after vestibular vertigo syndrome, but vestibular dysfunction did not have any effect on the further development of mental disorders^28^.

Our study showed that the proportion of PPPD patients complicated with emotional disorders was 46.72%, and the proportion of anxiety and depression was 3.1:1. Anxiety and depression were significantly higher in the patients with p-PPPD than in the patients with s-PPPD (52.33% vs. 30.56%; 16.28% vs. 11.11%, respectively). Clearly, anxiety disorder is common in patients with PPPD; a Chinese study showed that the incidence of anxiety disorders in PPPD was 48.57% and that of depression was 17.14%, and the longer the course of the disease was, the greater the probability of mental disease^29^. Studies have also shown that 42.5% of patients with organic dizziness have mental diseases, and these patients show more severe psychosocial disorders than patients without mental disorders^30^. A retrospective study showed that the proportion of PPPD patients with depression was 17.7%, while the proportion of patients with anxiety was 15.4%. However, the data were based on the medical history provided by the patients at admission and the evaluation of some neurologists; the patients were not evaluated by psychiatrists^10^. Due to the low consistency between the diagnosis of neurologists and the structured clinical interview of mental diseases (κ < 0.2), in our study, the prevalence of psychosocial disorders was based on the written or oral information provided by the patients themselves and the evaluation of special psychiatrists when necessary after admission. We still believe that clinical screening for psychiatric comorbidities in patients with PPPD is necessary.

## Data Availability

The datasets analysed during the current study are not publicly available, because we are analyzing the data and writing other relevant papers. But the datas are available from the corresponding author on reasonable request.
